# Vasodilator Activity of Compounds Isolated from Plants Used in Mexican Traditional Medicine

**DOI:** 10.3390/molecules23061474

**Published:** 2018-06-18

**Authors:** Francisco J. Luna-Vázquez, César Ibarra-Alvarado, María del Rayo Camacho-Corona, Alejandra Rojas-Molina, J. Isela Rojas-Molina, Abraham García, Moustapha Bah

**Affiliations:** 1 Laboratorio de Investigación Química y Farmacológica de Productos Naturales, Facultad de Química, Universidad Autónoma de Querétaro, C.P. 76010 Querétaro, Mexico; fjlunavz@yahoo.com.mx (F.J.L.-V.); rojasa@uaq.mx (A.R.-M.); jirojas@gmail.com (J.I.R.-M.); moubah@uaq.mx (M.B.); 2 Universidad Autónoma de Nuevo León, Facultad de Ciencias Químicas, Ciudad Universitaria, San Nicolás de los Garza, CP 66451 Nuevo León, Mexico; edgar.garciazp@uanl.edu.mx

**Keywords:** corosolic acid, 5,8,4′-trihydroxy-3,7-dimethoxyflavone, *meso*-dihydroguaiaretic acid hydrogen sulfide, nitric oxide, vasorelaxation

## Abstract

Arterial hypertension is one of the main risk factors in the development of cardiovascular diseases. Therefore, it is important to look for new drugs to treat hypertension. In this study, we carried out the screening of 19 compounds (triterpenes, diterpenes, sesquiterpenes, lignans, and flavonoids) isolated from 10 plants used in Mexican traditional medicine to determine whether they elicited vascular smooth muscle relaxation and, therefore, could represent novel anti-hypertension drug candidates. The vasorelaxant activity of these compounds was evaluated on the isolated rat aorta assay and the results obtained from this evaluation showed that three compounds induced a significant vasodilatory effect: *meso*-dihydroguaiaretic acid [half maximal effective concentration (EC_50_), 49.9 ± 11.2 µM; maximum effect (Emax), 99.8 ± 2.7%]; corosolic acid (EC_50_, 108.9 ± 6.7 µM; Emax, 96.4 ± 4.2%); and 5,8,4′-trihydroxy-3,7-dimethoxyflavone (EC_50_, 122.3 ± 7.6 µM; Emax, 99.5 ± 5.4%). Subsequently, involvement of the NO/cyclic guanosine monophosphate (cGMP) and H_2_S/ATP-sensitive potassium channel (K_ATP_) pathways on the vasodilator activity of these compounds was assessed. The results derived from this analysis showed that the activation of both pathways contributes to the vasorelaxant effect of corosolic acid. On the other hand, the vasodilator effect of *meso*-dihydroguaiaretic acid and 5,8,4′-trihydroxy-3,7-dimethoxyflavone, partly involves stimulation of the NO/cGMP pathway. However, these compounds also showed an important endothelium-independent vasorelaxant effect, whose mechanism of action remains to be clarified. This study indicates that *meso*-dihydroguaiaretic acid, corosolic acid, and 5,8,4′-trihydroxy-3,7-dimethoxyflavone could be used as lead compounds for the synthesis of new derivatives with a higher potency to be developed as drugs for the prevention and treatment of cardiovascular diseases.

## 1. Introduction

Arterial hypertension is considered a major risk factor in the development of several cardiovascular diseases, such as myocardial infarction and stroke [1,2,3], which together were responsible for 15 million deaths in 2015 [4]. Although several drugs are currently used in the treatment of high blood pressure, less than a third of the hypertension cases are successfully treated [5,6,7,8].

It has been broadly demonstrated that endothelial dysfunction, characterized by a diminished availability of endothelial relaxing factors, significantly contributes to the development of hypertension and thus, other cardiovascular diseases [9,10,11]. Particularly, reduced release of the gasotransmitters NO and H_2_S results in an impaired regulation of the vascular tone [12,13,14]. Therefore, the NO/cyclic guanosine monophosphate (cGMP) and H_2_S/ATP-sensitive potassium channel (K_ATP_) pathways are valuable targets for innovative treatments in hypertension [12,13,14].

In Mexico, plants are an important element of traditional medicine, and many of them are considered part of Mexican cultural heritage from prehispanic and colonial times [15,16]. Nevertheless, relatively few systematic scientific studies have been conducted to fully characterize the chemical composition and pharmacological activities of Mexican medicinal plants.

In the present study, we carried out a pharmacological screening of 19 secondary metabolites isolated from 10 plants used in Mexican traditional medicine in order to detect leads that could be developed as potential drug candidates to treat hypertension. The plant sources of the tested compouds were: *Amphipterygium adstringens* (Schltdl.) Standl. (*cuachalalate*), *Celaenodendron mexicanum* Standl. (*palo prieto*), *Crataegus gracilior* J.B. Phipps (*tejocote*), *Croton alamosanus* Rose (*vara blanca*), *Croton glabellus* L. (*cascarillo*), *Galphimia glauca* Cav. (*árnica roja*), *Jatropha neopauciflora Pax* (*sangre de grado*), *Larrea tridentata* (Sessé and Moc. ex DC.) Coville (*gobernadora*), *Perezia adnata* A. Gray (*pipitzáhuac*), and *Teloxys graveolens* (*epazote de zorrillo*). These plants are widely used in Mexican traditional medicine to treat several illnesses, such as gastric ulcers, stomach ache, gastroenteritis, diarrhea, dysentery, sores and infections of the oral cavity, skin ulcers [17,18,19,20,21], renal disorders and kidney stones (*Larrea tridentata*) [17], cough, asthma, tachycardia and to improve coronary blood flow (*Crataegus gracilior*) [20,22], and cancer (*Croton alamosanus*) [23].

**Figure 1:**
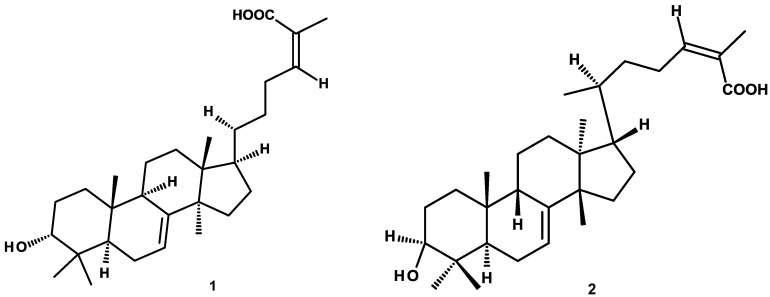

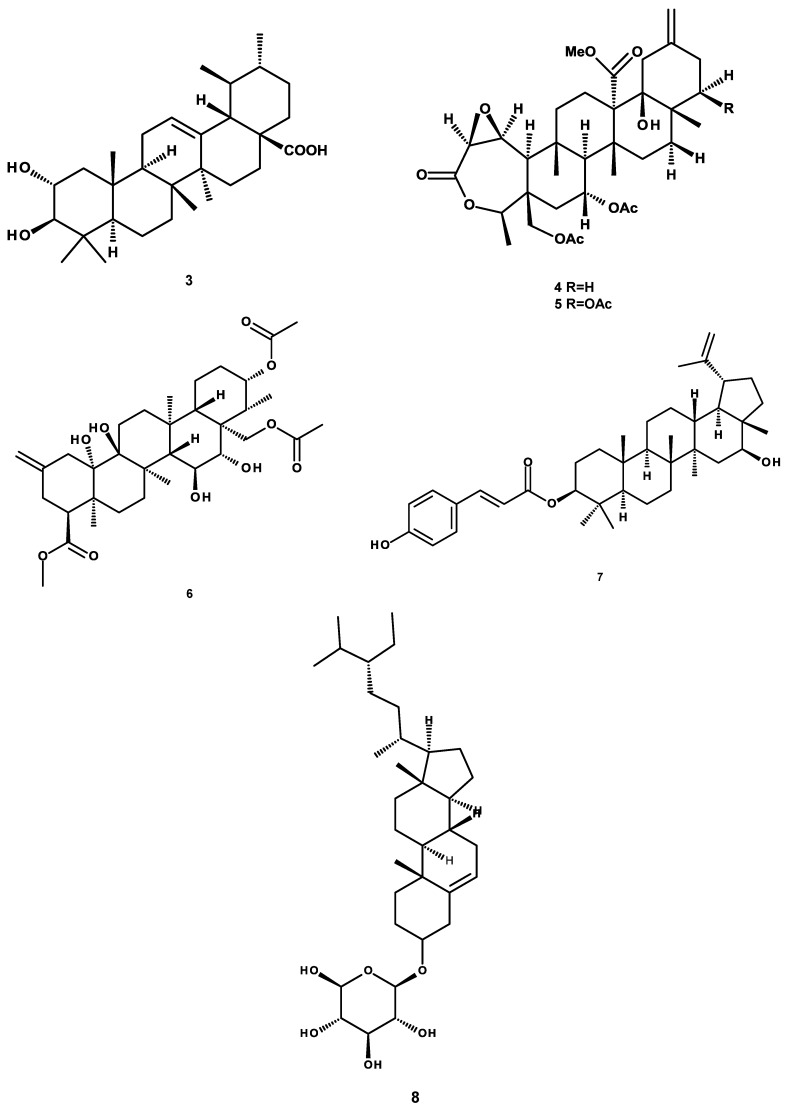

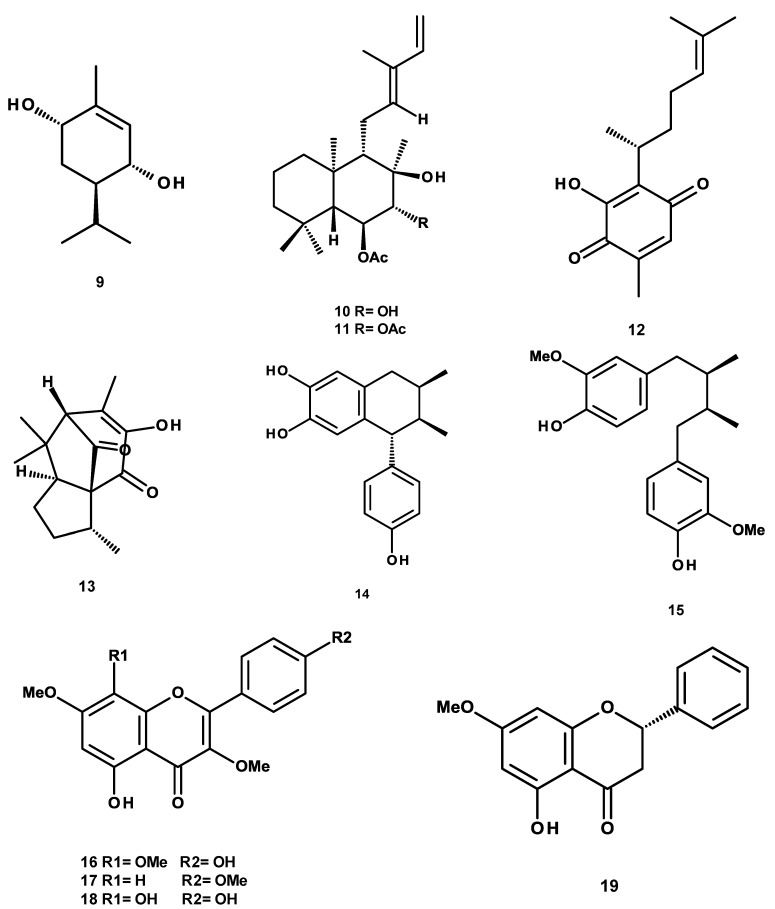
Chemical structures of studied compounds.

The compounds evaluated were previously purified by our research group and our collaborators [18,19,24,25,26] and include: (a) triterpenes: 3-α-hydroxymasticadienonic acid (**1**), 3α-hydroxytirucalla-7,22*Z*-dien-26-oic acid (**2**), corosolic acid (**3**), galphin A (**4**), galphin B (**5**), galphimidin (**6**), and 3β-*trans*-*p*-coumaroyl-oxy-16-β-hydroxy-20(29)-lupene (**7**); (b) sterols: β-sitosteryl β-d-glucopyranoside (**8**); (c) diterpenes: (3*R*,4*R*,6*S*)-*p*-menth-1-eno-3,6-diol (**9**), 6,7-diacetylaustro inulin (**10**), and 6-*O*-acetylaustro inulin (**11**); (d) sesquiterpenes: perezone (**12**), and pipitzol (**13**); (e) lignans: 3′-demethoxy-6-*O*-demethyl-isoguaiacin (**14**) and *meso*-dihydroguaiaretic acid (**15**); and (f) flavonoids: 5,4′-dihydroxy-3,7,8-trimethoxyflavone (**16**), 5-hydroxy-3,7,4′-trimethoxyflavone (**17**), 5,8,4′-trihydroxy-3,7-dimethoxyflavone (**18**), and pinostrobin (**19**) (Figure 1). Although it has been previously demonstrated that some of these compounds possess biological activities, such as antibacterial and antifungal [21,25,27], antiprotozoal [18,24,28,29], anti-inflammatory [30], anti-diabetic [31,32], and anticancer [23], at present, there are no studies aimed at assessing their vasodilatory activity.

In the first phase of this study, we assessed whether the selected compounds elicited vascular smooth muscle relaxation in the isolated rat aorta. Thereafter, the most potent vasodilator compounds, which also displayed the highest maximum effects [maximum effect (Emax) ≥ 96%], were evaluated to determine if the NO/cGMP and H_2_S/K_ATP_ channel pathways were involved in their mechanism of action.

## 2. Results

**Figure 2:**
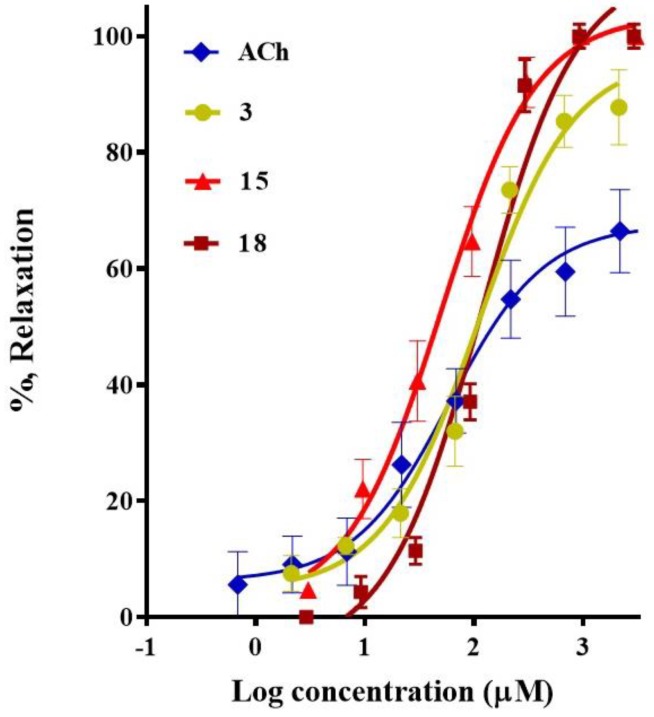
Concentration-response curves of the vasodilator effect of corosolic acid (**3**), *meso*-dihydroguaiaretic acid (**15**), and 5,8,4′-trihydroxy-3,7-dimethoxyflavone (**18**). Acetylcholine was used as a positive control.

The results from the pharmacological evaluation of the selected compounds indicated that 3-α-hydroxymasticadienonic acid (**1**), corosolic acid (**3**), galphimidin (**6**), *meso*-dihydroguaiaretic acid (**15**), 5,8,4′-trihydroxy-3,7-dimethoxyflavone (**18**), and pinostrobin (**19**) elicited a significant vasodilation. All these six secondary metabolites exhibited a maximum vasodilator effect up to 96%, which was higher than that of Acetylcholine (ACh), used as the positive control (Table 1). *meso*-Dihydroguaiaretic acid (**15**) exhibited a comparable potency to that of Ach, while corosolic acid (**3**) and 5,8,4′-trihydroxy-3,7-dimethoxyflavone (**18**) turned out to be approximately two-fold less potent than the positive control (Figure 2 and Table 1). Based on these results, involvement of the NO/cGMP and H_2_S/K_ATP_ channel pathways in the mechanisms underlying the vasorelaxant action of *meso*-dihydroguaiaretic acid (**15**) and 5,8,4′-trihydroxy-3,7-dimethoxyflavone (**18**), both isolated from *Larrea tridentata*, and corosolic acid (**3**) purified from *Crataegus gracilior*, was analyzed.

### 2.1. Participation of the Endothelium in the Vasorelaxant Response of Compounds ***3***, ***15***, and ***18***

**Figure 3:**
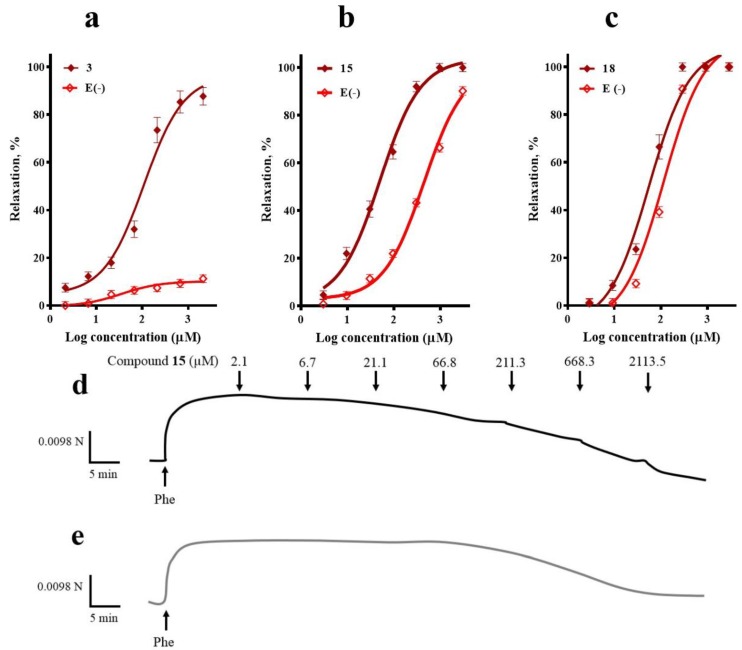
Concentration-response curves of the vasodilator effect of (**a**) corosolic acid (**3**), (**b**) *meso*-dihydroguaiaretic acid (**15**), and (**c**) 5,8,4′-trihydroxy-3,7-dimethoxyflavone (**18**) in the presence and absence (E-) of endothelium. A typical trace in which *meso*-dihydroguaiaretic acid (**15**) inhibited Phe-induced contraction in endothelium-intact (**d**), and -denuded aorta (**e**). Analysis by an unpaired *t*-test was made to test for differences between EC_50_s of each compound in the presence and absence of endothelium (**3**, *p* < 0.0001; **15**, *p* < 0.0001; **18**, *p* = 0.0093).

Figure 3 shows the concentration-response curves (CRCs) for corosolic acid (**3**), *meso*-dihydroguaiaretic acid (**15**), and 5,8,4′-trihydroxy-3,7-dimethoxyflavone (**18**) in the presence and absence of endothelium.

The vasorelaxation induced by corosolic acid (**3**) was almost completely blocked in the absence of endothelium, which suggested that endothelial factors mediate its vasodilatory effect. On the other hand, endothelial denudation caused a significant rightward shift of the CRC to *meso*-dihydroguaiaretic acid (**15**) and 5,8,4′-trihydroxy-3,7-dimethoxyflavone (**18**), indicating the contribution of both endothelium-independent and dependent pathways in their mechanism of action.

### 2.2. Participation of the NO/cGMP and H_2_S/K_ATP_ Channel Pathways in the Vasodilator Response of Compounds ***3***, ***15***, and ***18***

**Figure 4:**
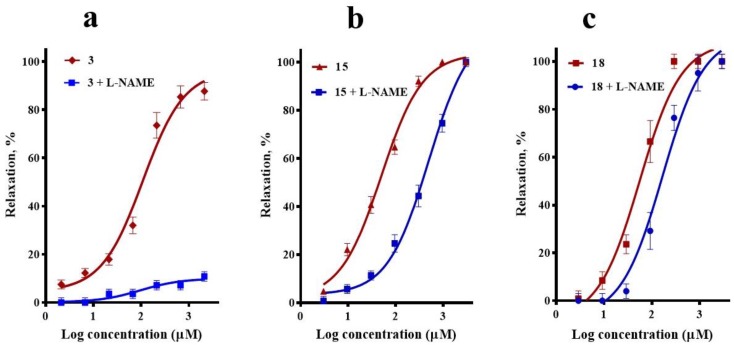
Vasodilatory effect of (**a**) corosolic acid (**3**), (**b**) *meso*-dihydroguaiaretic acid (**15**), and (**c**) 5,8,4′-trihydroxy-3,7-dimethoxyflavone (**18**) in the absence (control) and presence of L-NAME (100 µM). Analysis by one-way analysis of variance (ANOVA) was made between the curves of each compound in the absence and presence of N^G^-nitro-l-arginine methyl ester (L-NAME) followed by a post hoc Tukey’s test (**3**, *p* < 0.0001; **15**, *p* = 0.006; **18**, *p* = 0.991).

The effect of N^G^-nitro-l-arginine methyl ester (L-NAME, 100 µM), an inhibitor of endothelial nitric oxide synthase, on the vasorelaxation induced by compound **3** closely resembled that which occurred in the absence of endothelium, clearly supporting that activation of the NO/cGMP pathway importantly contributes to corosolic acid (**3**)-vasodilation. Regarding *meso*-dihydroguaiaretic acid (**15**)-induced vasodilation, it was significantly reduced (*p* = 0.006), but not completely blocked, in the presence of L-NAME, indicating that the effect of this lignan partly depends on stimulation of the NO/cGMP pathway. On the other hand, the inhibition of endothelial nitric oxide synthase (eNOS) with L-NAME produced a slight rightward shift of the CRC of 5,8,4′-trihydroxy-3,7-dimethoxyflavone (**18**) (*p* = 0.99) (Figure 4).

**Figure 5:**
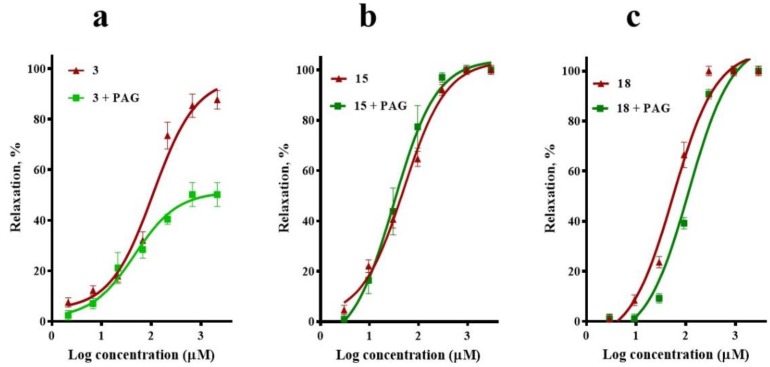
Vasodilatory effect of (**a**) corosolic acid (**3**), (**b**) *meso*-dihydroguaiaretic acid (**15**), and (**c**) 5,8,4′-trihydroxy-3,7-dimethoxyflavone (**18**) in the absence (control) and presence of propargylglycine (PAG, 1 mM). Analysis by one-way ANOVA was made between the curves of each compound in the absence and presence of PAG followed by a post hoc Tukey’s test (**3**, *p* = 0.001; **15**, *p* = 0.803; **18**, *p* = 0.102).

To determine whether the H_2_S/K_ATP_ pathway participated in the vasodilatory effect of compounds **3**, **15**, and **18**, the effect of propargylglycine (PAG; 1 mM), an inhibitor of cystathionine gamma-lyase (CSE), was assessed. This inhibitor significantly decreased the maximum vasodilator effect of corosolic acid (**3**) from ~97% to ~50%, which supported the idea that the H_2_S/K_ATP_ channel pathway is also involved in the vasorelaxant mechanism of this compound. By contrast, the presence of PAG did not significantly modify the CRCs of *meso*-dihydroguaiaretic acid (**15**) and 5,8,4′-trihydroxy-3,7-dimethoxyflavone (**18**), discarding involvement of the H_2_S/K_ATP_ pathway in their effect (Figure 5).

### 2.3. Involvement of K^+^ Channels in the Vasodilation Evoked by Compounds ***3***, ***15***, and ***18***

**Figure 6:**
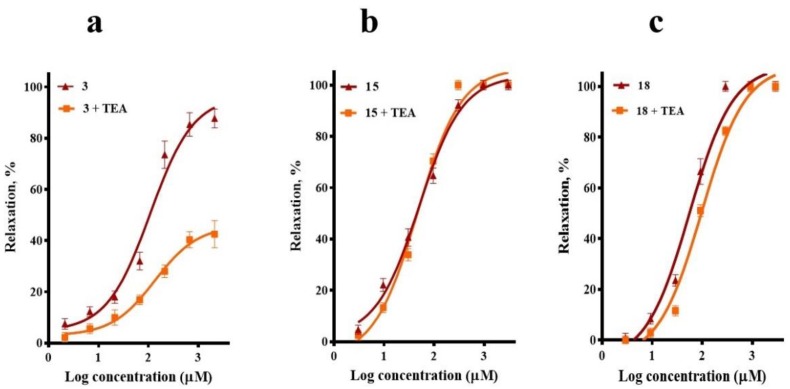
Vasodilatory effect of (**a**) corosolic acid (**3**), (**b**) *meso*-dihydroguaiaretic acid (**15**), and (**c**) 5,8,4′-trihydroxy-3,7-dimethoxyflavone (**18**) in the absence (control) and presence of tetraethylammonium (TEA, 10 mM). Analysis by one-way ANOVA was made between the curves of each compound in the absence and presence of TEA followed by a post hoc Tukey’s test (**3**, *p* < 0.0001; **15**, *p* = 0.868; **18**, *p* = 0.429).

To test whether the activation of K^+^ channels was implicated in the vasodilatory effect of compounds **3**, **15**, and **18**, tetraethylammonium (TEA) was used to block K^+^ channels. TEA pre-treatment significantly reduced the corosolic acid (**3**)-vasorelaxant effect, without significantly altering the CRCs of *meso*-dihydroguaiaretic acid (**15**) and 5,8,4′-trihydroxy-3,7-dimethoxyflavone (**18**) (Figure 6).

## 3. Discussion

In the present study, we carried out the pharmacological evaluation of 19 natural products, which we had previously isolated from 10 plants widely used in Mexican traditional medicine, in the search of leads that could be used to develop new therapeutic agents to treat cardiovascular diseases.

All compounds tested caused a concentration-related relaxation of l-phenylephrine-contracted rat aortic rings. It is important to highlight that 3-α-hydroxymasticadienonic acid (**1**), corosolic acid (**3**), galphimidin (**6**), *meso*-dihydroguaiaretic acid (**15**), 5,8,4′-trihydroxy-3,7-dimethoxyflavone (**18**), and pinostrobin (**19**) exhibited a maximum vasodilator effect higher than that of acetylcholine. Of these six compounds, the most potent were corosolic acid (**3**), *meso*-dihydroguaiaretic acid (**15**), and 5,8,4′-trihydroxy-3,7-dimethoxyflavone (**18**), which belong to structural classes of natural products that have shown vasodilatory effects [33].

Considering that stimulation of the NO/cGMP and H_2_S/K_ATP_ pathways in endothelial cells importantly contributes to vasorelaxation, we investigated whether the presence of endothelium and activation of these biochemical pathways were involved in the vasorelaxant effect elicited by compounds **3**, **15**, and **18**.

Earlier studies have proved that naturally occurring lupane-, oleanane-, and ursane-type triterpenes elicit vasodilator effects [34,35,36,37,38]. The vasodilation produced by the lupane-type triterpene betulinic acid and the oleanane-type triterpenes moronic, morolic, and 3,4-seco-olean-18-enen-3,28-dioic acids is endothelium-dependent [35]. While, according to different scientific groups, the vasorelaxation evoked by oleanolic acid might be either nearly fully endothelium-dependent or endothelium-dependent and independent [36,38], the cause of this discrepancy has been attributed to the compound employed to pre-contract the vessels, phenylephrine [38] or norepinephrine [36].

In the case of the present study, blockade of the vasodilator activity produced by corosolic acid (**3**) in endothelium-denuded aortas provided evidence that the mechanism underlying this triterpene-induced vasodilation was endothelium-dependent. Supporting this finding, the inhibition of both eNOS and CSE significantly decreased corosolic acid-induced vasodilation, indicating the participation of the NO/cGMP and the H_2_S/K_ATP_ pathways in its effect. These signaling pathways activate different potassium channels, subsequently hyperpolarizing the vascular smooth muscle cell membrane, leading to a decrease in the intracellular free calcium level, which finally produces vasodilation [39,40,41,42,43,44]. In this study, we evaluated whether potassium channel blockers impaired vasodilation provoked by compound **3**. TEA significantly diminished corosolic acid-evoked vasodilation, which confirmed the activation of signaling pathways for NO and H_2_S.

Corosolic acid (**3**) is an ursane-type triterpene, which has a similar structure to that of ursolic acid but with an alpha OH group at position 2. Interestingly, our research group previously demonstrated that ursolic acid and uvaol (the alcohol corresponding to ursolic acid) produce an endothelium dependent vasorelaxation, which involves stimulation of the NO/cGMP and H_2_S/K_ATP_ pathways. Molecular docking studies showed that both triterpenes are able to bind with high affinity to endothelial NOS and CSE in allosteric binding sites located relatively far from the catalytic sites [34]. We found that corosolic acid (**3**) (EC_50_ = 108.9 ± 3.2 µM; E_max_ = 96.4 ± 4.2%) is approximately two fold less potent than ursolic acid (EC_50_ = 47.1 ± 7.6 µM; E_max_ = 97.7 ± 3.9%) and uvaol (EC_50_ = 43.6 ± 5.6 µM; E_max_ = 93.4 ± 5.1%) [34]. However, these three compounds exhibited a similar maximum vasodilator effect. Considering the great structure resemblance between ursolic acid and corosolic acid (**3**), it is very likely that this latter compound directly activates eNOS and CSE in a similar way to ursolic acid. Both compounds bear a β-OH group at position 3 and an α-carboxyl group at position 28. According to the molecular docking studies of ursolic acid, 3 β-OH forms hydrogen bonds with Asp 480 in eNOS and His 99 in CSE. Whereas, 28 α-carboxyl forms a hydrogen bond with His373 and an electrostatic interaction with Arg367 in eNOS [34]. All these interactions are key in stabilizing protein-ligand binding. It is quite possible that these same types of interactions are established between corosolic acid (**3**) and its enzymatic targets.

In a recent study, Waldbauer et al. carried out a bioassay-guided fractionation of the methanol/water (70:30) extract form apple pomace, employing the ^14^C-l-arginine to ^14^C-l-citrulline conversion assay, in a human endothelium cell line, to monitor eNOS activity. Eleven triterpenoid acids, including ursolic and corosolic acids, were isolated from the most active fractions. None of the individual triterpenes increased eNOS activity, but the reconstituted compound mixture did significantly stimulate eNOS [45]. It is important to mention that the range of concentrations of ursolic (1 to 7.5 µM) and corosolic (1 to 15 µM) acids employed to test the activation of eNOS in endothelial cells was lower than in the current study (2.1 µM to 2.1 mM), which may be the reason why Waldbauer et al. did not observe any effect on eNOS.

Several studies have been reported about the biological activities elicited by corosolic acid (**3**), including anti-inflammatory [30], anti-obesity [46], antitumoral [47], and antidiabetic effects [31,32]. However, its action on vascular smooth muscle has not so far been investigated. Yamaguchi et al. (2006) reported that corosolic acid (0.072%), administered in a high fat diet during 14 weeks, lowered blood pressure in male spontaneously hypertensive corpulent rat (SHR-cp) rats, an animal model of metabolic syndrome [48], but the molecular mechanism underlying the hypotensive effect of this triterpene was not clarified. Corosolic acid-induced vasodilator activity may account for the anti-hypertensive properties observed in the SHR-cp rats.

Although NO and H_2_S independently elicit a smooth muscle relaxation, there is growing evidence that interaction between the NO/cGMP and H_2_S/K_ATP_ pathways leads to an enhancement of the vasorelaxant effect [49], through various mechanisms, which include: stimulation of eNOS activity, stabilization of soluble guanylate cyclase, inhibition of phosphodiesterases, activation of protein kinases [12,49,50], and generation of nitroxyl (HNO), a potent vasodilator [51,52,53]. Since corosolic acid (**3**) activates both pathways, it might be considered a valuable lead compound for the development of new drugs useful for the treatment of cardiovascular diseases related to endothelial dysfunction.

Regarding *meso*-dihydroguaiaretic acid (**15**), a variety of biological activities, such as antioxidant [54], anti-inflammatory [55], cytotoxic, and antineoplasic [56,57] effects, have been ascribed to this compound. Concerning the cardiovascular system, it has been documented that this lignan is a moderate platelet-activating factor (PAF) antagonist [58] and an inhibitor of vascular smooth muscle cell proliferation [59]. The present study provides heretofore unknown evidence that compound 15 is capable of inducing vasodilation, via mechanisms that involve endothelium-dependent and independent pathways. The endothelium-dependent component is mainly produced by activation of the NO/cGMP pathway.

Previous studies demonstrated that nordihydroguaiaretic acid, a lignan structurally related to 15, which instead of possessing methoxyl substituents in positions 3 and 3′ has hydroxyl groups, enhances the expression and activity of eNOS in endothelial cells [60] and activates large conductance calcium-dependent potassium (BK) channels in arterial smooth muscle cells [61]. It has been proposed that BK channels opening by nordihydroguaiaretic acid is produced by a direct action on the BK alfa subunit and an increased calcium release from the sarcoplasmic reticulum [62]. In contrast to these findings, our results showed that activation of the potassium channels is not involved in the vasodilator effect elicited by *meso*-dihydroguaiaretic acid (**15**). The reason for this difference might be attributed to the presence of methoxy groups on carbons 3 and 3′ in compound 15. In accordance with this hypothesis, it has been suggested that the presence of the hydroxy group in position 5 in 5-hydroxyflavone is a structural requirement for a possible interaction with BK channels [63].

Considering that stimulation of the potassium channels is not implicated in the vasorelaxant mechanism of *meso*-dihydroguaiaretic acid (**15**), it is possible that activation of the NO/cGMP pathway induced by this lignan produces vasodilation through alternative mechanisms, including the inhibition of calcium influx from voltage-dependent calcium channels and calcium release by the inositol 1,4,5-trisphosphate receptor or increase of the reuptake of cytosolic calcium into the sarcoplasmic reticulum [64]. It is worth highlighting that compound 15 elicited a vasodilator effect with a similar potency to that of ACh, which supports its potential value as a lead compound for the development of new antihypertensive drugs.

On the other hand, the present study represents the first demonstration that 5,8,4′-trihydroxy-3,7-dimethoxyflavone (**18**) is capable of relaxing the arterial smooth muscle. Removal of endothelium significantly decreased, but not completely blocked, the vasorelaxation induced by this flavone, indicating that both endothelial-dependent and independent vasodilation pathways are involved in its mechanism of action. The vasorelaxing effect was significantly diminished in the presence of L-NAME, which evidenced that activation of the NO/cGMP pathway partly contributes to endothelium dependent 5,8,4′-trihydroxy-3,7-dimethoxyflavone-induced vasodilation. Considering the slight rightward shift of the CRCs in the absence of endothelium (*p* = 0.009) and in the presence of L-NAME (*p* = 0.99), it is evident that this compound mainly produces its vasodilator effect through an endothelium-independent mechanism. Our results are in accordance with previous studies which proved that methoxy-flavones, structurally related to compound **18**, elicit vasodilation [65,66], at least partly, through an endothelium-dependent mechanism that involves activation of the NO/cGMP pathway [65]. However, our findings differ from those of other researchers who found that certain methoxy-flavones produce an endothelium-dependent vasodilatory effect via activation of the potassium channels [67,68]. Apparently, differences in the type and position of the substituents on the basic flavone skeleton greatly influence the mechanism underlying the vasodilator effect of these compounds, as evidenced by the fact that 5,4′-dihydroxy-3,7,8,3′-tetramethoxyflavone produces an endothelium-independent vasorelaxation, mediated by potassium channels activation [69], whereas 3,5,7,3′,4′-pentamethoxyflavone induces a vasorelaxant effect, which provokes the release of NO and H_2_S, but surprisingly without activating potassium channels [70,71]. In the case of 5,8,4′-trihydroxy-3,7-dimethoxyflavonen (**18**), our results indicate that this compound mainly produces its vasodilator effect through an endothelium-independent mechanism, which may involve a calcium channel blockade [72,73]. However, this remains to be elucidated.

## 4. Materials and Methods

### 4.1. Reagents and Chemicals

Reagents, standards, and solvents used in the pharmacological assays were obtained from Sigma-Aldrich (St. Louis, MO, USA).

### 4.2. Isolation, Purification and Structural Characterization of Phytochemicals

The phytochemicals evaluated were previously isolated and purified by several chromatographic methods from different plants. The purity of the compounds was determined by one dimension or two-dimension thin layer chromatography and in some cases using high performance liquid chromatography. The chemical structure of compounds was determined by 1D or 2D Nuclear Magnetic Resonance techniques as described previously: *A. adstringens*: 3-α-hydroxymasticadienonic acid (**1**) [74], *C. mexicanum*: 3α-hydroxytirucalla-7,22*Z*-dien-26-oic acid (**2**) [18], *C. gracilior*: corosolic acid (**3**) [26], *C. alamosanus*: (3*R*,4*R*,6*S*)-*p*-menth-1-eno-3,6-diol (**9**), 5- hydroxy-3,7,4′-trimethoxyflavone (**17**), *C. glabellus*: 6,7-diacetylaustro inulin (**10**), and 6-*O*-acetylaustro inulin (**11**) [75], *G. glauca*: galphin A (**4**), galphin B (**5**), galphimidin (**6**) [24], *J. neopauciflora*: 3β-*trans*-*p*-coumaroyl-oxy-16-β-hydroxy-20(29)-lupene (**7**), β-sitosteryl β-d-glucopyranoside (**8**) [19], *L. tridentata*: 3′-demethoxy-6-*O*-demethyl-isoguaiacin (**14**), *meso*-dihydroguaiaretic acid (**15**), 5,4′-dihydroxy-3,7,8-trimethoxyflavone (**16**), 5,8,4′-trihydroxy-3,7-dimethoxyflavone (**18**) [25], *P. adnata*: perezone (**12**), and pipitzol (**13**) [76], and *T. graveolens*: pinostrobin (**19**) [28].

### 4.3. Experimental Animals

All experimental procedures were performed in accordance with guidelines of the Mexican Official Standard NOM-062-ZOO-1999 [74], and approved by the Bioethics Committee of the Deparment of Postgraduate, Research and Innovation of the Autonomous University of Querétaro, México (Approval number 290/SPII/2018). Wistar male rats (250–300 g) were provided by the Institute of Neurobiology of the National Autonomous University of Mexico, Campus Juriquilla. Animals were housed in standard cages under controlled temperature conditions with a 12:12 h light-dark cycle. Water and food were provided ad libitum.

### 4.4. Determination of the Vasodilator Effect of the Selected Secondary Metabolites

#### 4.4.1. Isolated Rat Aorta Assay

Rats were sacrificed by decapitation. The thoracic aorta was surgically removed and placed in a Petri dish containing ice-cold (4 °C) Krebs-Henseleit solution with the following composition (mM): 126.8 NaCl; 5.9 KCl; 1.2 KH_2_PO_4_; 1.2 MgSO_4_; 5.0 d-glucose; 30 NaHCO_3_; 2.5 CaCl_2_ (pH 7.4), bubbled with a mixture of carbogen (95% O_2_ and 5% CO_2_). Then, the intraluminal space of the aorta was rinsed with fresh solution to prevent clot formation, cleaned from surrounding connective tissue, and sliced into rings (3–4 mm in length). Aortic rings were mounted between two metallic hooks, with one being fixed and the other attached to an isometric transducer, and placed into organ baths chambers containing pre-warmed Krebs-Henseleit solution (37 °C) gassed with carbogen. The aortic segments were allowed to equilibrate for 60 min under a resting tension of 1.5 g. During the resting period, the organ bath solution was replaced every 10 min. In order to stimulate the vascular smooth muscle, the tissues were contracted with KCl solution (100 mM). Once a stable contractile tone had been reached, the bathing medium was replaced every 10 min to restore the initial resting tension. Afterwards, the aortic rings were contracted with 1 µM l-phenylephrine (Phe); the contractile force induced was defined as 100%, and once the plateau was reached, the test compounds were cumulatively added. Acetylcholine was dissolved in distilled water, while the selected compounds were dissolved in dimethyl sulfoxide (DMSO) and diluted in distilled water. The highest concentration of DMSO was 0.2% (*v*/*v*). When used, pharmacological inhibitors were added to the organ bath chambers 20 min before the addition of Phe. Changes in tension caused by the tested concentrations were detected by Grass FT03 force transducers coupled to a Grass 7D Polygraph (Grass Instrument Co, Quincy, MA, USA); they were expressed as percentages of relaxation based on the contraction generated by adding Phe [77].

#### 4.4.2. Participation of the Endothelium in the Vasorelaxant Response of Compounds **3**, **15**, and **18**

To determine whether the vasorelaxant response of compounds **3**, **15**, and **18** was dependent on the vascular endothelium, assays on aorta segments without endothelium were performed. In these experiments, the endothelium was removed by flushing the lumen of the aorta with 0.2% desoxycholic acid in saline solution 0.9%, as previously reported [77]. The absence of endothelium was confirmed at the start of the experiments, showing that the addition of 1 μM of ACh did not induce more than 5% relaxation.

### 4.5. Evaluation of the Participation of the NO/cGMP and H_2_S/K_ATP_ Channel Pathways in the Vasodilator Response of Compounds ***3***, ***15***, and ***18***

Involvement of the NO/cGMP and the H_2_S/K_ATP_ channel pathways in the vasodilator effect of compounds **3**, **15**, and **18** was evaluated by incubating intact endothelium aortic rings for 20 min in the presence of inhibitors of specific enzymes of each of these pathways: (1) NO/cGMP pathway: 100 μM N^G^-nitro-l-arginine methyl ester (L-NAME, inhibitor of eNOS); and (2) H_2_S/K_ATP_ channel pathway: 10 mM dl-propargylglycine (PAG, inhibitor of CSE) [34,78,79].

To assess whether activation of the K^+^ channels was involved in the vasodilation produced by compounds **3**, **15**, and **18**, the effect of pretreatment with the non-selective potassium channel blocker, 1 mM TEA, was evaluated [80,81].

### 4.6. Statistical Analysis

Evaluations of each concentration of the tested substances were performed on aortas obtained from at least three different rats (*n* = 3) with two replicas.

All values are expressed as the mean ± standard error of the mean (SEM). The resulting data obtained from each evaluation were fitted to a sigmoidal equation, plotted, and analyzed to calculate EC_50_ and Emax (GraphPad Prism 7.02, San Diego, CA, USA).

In the case of endothelium-dependent evaluations, analysis by an unpaired *t*-test was conducted to test differences between the EC_50_s of each compound in the presence and absence of endothelium. While CRC in the presence of inhibitors was analyzed by one-way analysis of variance (ANOVA) followed by the Turkey test to evaluate any significant differences between the means. Values of *p* < 0.01 were considered to be significant.

## 5. Conclusions

This study demonstrates that 19 natural products obtained from plants widely employed in Mexican traditional medicine are able to modify the tone of arterial smooth muscle. The most potent vasodilatory compounds were corosolic acid (**3**), *meso*-dihydroguaiaretic acid (**15**), and 5,8,4′-trihydroxy-3,7-dimethoxyflavone (**18**). These secondary metabolites represent valuable leads for the development of drugs useful in the treatment of cardiovascular diseases. Corosolic acid induces vasodilation by a mechanism that involves activation of the NO/cGMP and H_2_S/K_ATP_ pathways. Additionally, the vasodilator effect of *meso*-dihydroguaiaretic acid and 5,8,4′-trihydroxy-3,7-dimethoxyflavone partly involves stimulation of the NO/cGMP pathway. However, these compounds also showed an important endothelium-independent vasorelaxant effect, whose mechanism of action remains to be clarified. Our findings confirm that plants traditionally used with medicinal purposes constitute an important reservoir of bioactive compounds that deserves intensive scientific exploration.

## Figures and Tables

**Table 1:** Values of half maximal effective concentration (EC_50_) and maximum effect (Emax) of positive control (Acetilcholine, ACh) and test compounds.

Plant/Compound	EC_50_ (μM)	E_max_ (%)
acetilcholine (ACh)	58.8 ± 8.9	69.5 ± 5.7
*Amphypterygium adstringens*		
3-α-hydroxymasticadienonic acid (**1**)	206.1 ± 11.6	98.2 ± 3.1
*Celaenodendron mexicanum*		
3α-hydroxytirucalla-7,22*Z*-dien-26-oic acid (**2**)	331.3 ± 42.1	99.5 ± 6.1
*Crataegus gracilior*		
Corosolic acid (**3**)	108.9 ± 6.7	96.4 ± 4.2
*Croton alamosanus*		
5-hydroxy-3,7,4′-trimethoxyflavone (**17**)	377.1 ± 37.1	80.5 ± 3.7
(3*R*,4*R*,6*S*)-*p*-menth-1-ene-3,6-diol (**9**)	1622.8 ± 73.8	99.5 ± 8.3
*Croton glabellus*		
6-*O*-acetylaustro inulin (**10**)	413.5 ± 22.4	47.1 ± 2.8
6,7-diacetylaustro inulin (**11**)	261.0 ± 9.1	99.5 ± 3.8
*Galphimia glauca*		
Galphin A (**4**)	592.3 ± 21.7	99.5 ± 9.1
Galphin B (**5**)	1030.7 ± 39.4	99.5 ± 23.2
Galphimidin (**6**)	145.9 ± 9.2	99.5 ± 5.3
*Jatropha neopauciflora*		
3β-*trans*-*p*-coumaroyl-oxy-16-β-hydroxy-20(29)-lupene (**7**)	63.2 ± 5.8	27.5 ± 1.9
β-sitosteryl β-d-glucopyranoside (**8**)	314.7 ± 19.7	71.2 ± 5.3
*Larrea tridentata*		
*meso*-dihydroguaiaretic acid (**15**)	49.9 ± 11.2	99.8 ± 2.7
5,4′-dihydroxy-3,7,8-trimethoxyflavone (**16**)	587.8 ± 33.4	80.5 ± 5.6
5,8,4′-trihydroxy-3,7-dimethoxyflavone (**18**)	122.3 ± 7.6	99.5 ± 5.4
3′-demethoxy-6-*O*-demethyl-isoguaiacin (**14**)	604.5 ± 60.1	91.70 ± 7.3
*Perezia adnata*		
perezone (**12**)	524.5 ± 32.5	99.5 ± 2.2
pipitzol (**13**)	249.4 ± 10.2	59.8 ± 2.4
*Teloxys graveolens*		
pinostrobin (**19**)	234.9 ± 9.9	99.5 ± 4.8
